# Phylogeography of Human and Animal *Coxiella burnetii* Strains: Genetic Fingerprinting of Q Fever in Belgium

**DOI:** 10.3389/fcimb.2020.625576

**Published:** 2021-02-26

**Authors:** Sara Tomaiuolo, Samira Boarbi, Tiziano Fancello, Patrick Michel, Damien Desqueper, Fabien Grégoire, Jozefien Callens, David Fretin, Bert Devriendt, Eric Cox, Marcella Mori

**Affiliations:** ^1^ Zoonoses of Animals Unit, Veterinary Bacteriology, Infectious Diseases in Animals Scientific Directorate, Sciensano, Brussels, Belgium; ^2^ Belgian Reference Centrum for Coxiella burnetii and Bartonella, Brussels, Belgium; ^3^ Laboratory of Immunology, Department of Virology, Parasitology and Immunology, Faculty of Veterinary Medicine, Ghent University, Merelbeke, Belgium; ^4^ Serology and Molecular Biology Unit, Association Régionale de Santé et d’Identification Animales (Arsia), Ciney, Belgium; ^5^ Small Ruminant Section, Dierengezondheidszorg (DGZ), Torhout, Belgium

**Keywords:** *Coxiella burnetii*, MLVA, SNP, WGS, humans, animals, alpaca

## Abstract

Q fever is a zoonotic disease caused by the bacteria *Coxiella burnetii.* Domestic ruminants are the primary source for human infection, and the identification of likely contamination routes from the reservoir animals the critical point to implement control programs. This study shows that Q fever is detected in Belgium in abortion of cattle, goat and sheep at a different degree of apparent prevalence (1.93%, 9.19%, and 5.50%, respectively). In addition, and for the first time, it is detected in abortion of alpaca (*Vicugna pacos*), raising questions on the role of these animals as reservoirs. To determine the relationship between animal and human strains, Multiple Locus Variable-number Tandem Repeat Analysis (MLVA) (n=146), Single-Nucleotide Polymorphism (SNP) (n=92) and Whole Genome Sequencing (WGS) (n=4) methods were used to characterize samples/strains during 2009-2019. Three MLVA clusters (A, B, C) subdivided in 23 subclusters (A1-A12, B1-B8, C1-C3) and 3 SNP types (SNP1, SNP2, SNP6) were identified. The SNP2 type/MLVA cluster A was the most abundant and dispersed genotype over the entire territory, but it seemed not responsible for human cases, as it was only present in animal samples. The SNP1/MLVA B and SNP6/MLVA C clusters were mostly found in small ruminant and human samples, with the rare possibility of spillovers in cattle. SNP1/MLVA B cluster was present in all Belgian areas, while the SNP6/MLVA C cluster appeared more concentrated in the Western provinces. A broad analysis of European MLVA profiles confirmed the host-species distribution described for Belgian samples. *In silico* genotyping (WGS) further identified the spacer types and the genomic groups of *C. burnetii* Belgian strains: cattle and goat SNP2/MLVA A isolates belonged to ST61 and genomic group III, while the goat SNP1/MLVA B strain was classified as ST33 and genomic group II. In conclusion, Q fever is widespread in all Belgian domestic ruminants and in alpaca. We determined that the public health risk in Belgium is likely linked to specific genomic groups (SNP1/MLVA B and SNP6/MLVA C) mostly found in small ruminant strains. Considering the concordance between Belgian and European results, these considerations could be extended to other European countries.

## Introduction

Q fever is a zoonosis caused by *Coxiella burnetii*, an intracellular gram-negative bacterium. Phylogenetic studies based on 16S rRNA classified *C. burnetii* within the Legionellas order of the γ-Proteobacteria (Stein et al., 1993). Although *C. burnetii* is able to infect a wide range of host species (Cutler et al., 2007), the primary animal reservoirs relevant for human infection are domestic ruminants (cattle, goat, sheep). In infected ruminants, the main symptoms are reproduction disorders along with abortion, stillbirth and infertility (To et al., 1998; Woldehiwet, 2004; Agerholm, 2013). Excretion of the microorganisms occurs massively during abortion, with the release of billions of bacteria in the placenta and birth fluids. The bacteria are also secreted *via* milk, feces and vaginal discharges after parturition for a variable time depending on the ruminant species (Berri et al., 2001; Arricau Bouvery et al., 2003; Guatteo et al., 2006; Rodolakis et al., 2007; Rousset et al., 2009). An additional source of human infection, especially in remote areas, might be represented by exposure to wild animals through tick bites or direct contact with wildlife (Jado et al., 2012; González-Barrio et al., 2016a).

Humans are easily infected through inhalation of contaminated aerosols (Maurin and Raoult, 1999). In most cases the pathology presents no or flu-like symptoms that may clear up in a few weeks, while in other cases (1%–5% of chronic manifestations) the infection can lead to serious life-threatening issues. Therefore, it is crucial to track and mitigate transmission routes from primary animal infection sources to humans. Current epidemiological data indicate human outbreaks related largely to goat and sheep infections (Roest et al., 2011; Georgiev et al., 2013).

Given the tight connection between ruminants’ infection and the risk of human exposure, it is important to establish the relationship between animal and human strains, to be able to identify the likely risk for public health. Molecular typing allows to identify strain-host interconnections and to determine transmission dynamics, useful for the development of targeted intervention or prevention strategies (Riley and Blanton, 2018; Riley, 2019). Nevertheless, *C. burnetii* genotyping is challenging due to the difficulties in obtaining animal, but especially human samples suitable for the genetic characterization. Considering the complexity of Q fever direct diagnosis and the low chronicity rate, human samples positive for *C. burnetii* are extremely limited, except during outbreak episodes. In addition, the inefficient and difficult culturing of *C. burnetii* strains from clinical or animal samples impedes the direct detection of these pathogens by other methods than PCR.

For the molecular characterization of *C. burnetii*, a harmonized reference method is yet to be designed. Initially, *C. burnetii* isolates were classified in six distinct genomic groups (GG I-VI) *via* DNA fingerprinting (Hendrix et al., 1991). Nowadays, this technique is substituted by more discriminant and reproducible methods, even though the Hendrix GG classification has never been abandoned. In fact, GG are also predicted from recent results obtained with newer methods (i.e. MST, ParSNP, CanSNP, MLVA, microarray) (Beare et al., 2006; Hornstra et al., 2011; Karlsson et al., 2014; Piñero et al., 2015; Hemsley et al., 2019). The GG I includes strains from tick, cattle and humans, while group II and IV are found in humans and small ruminants. GG III, V and VI are the most species-specific as they comprehend isolates from big ruminants (GG III), humans (GG V) and rodents (GG VI) (Hendrix et al., 1991; Hemsley et al., 2019). In 2006, two novel genetic groups, GG VII and GG VIII, were identified (Beare et al., 2006). The GG VII consisted of wild animal strains, and the GG VIII included strains that originated from small ruminant and human samples (Jado et al., 2012; González-Barrio et al., 2016b).

Today, the most adopted methods to define phylogeny are the multi loci variable-number tandem repeat analysis (MLVA) and the multispacer sequence typing (MST) (Arricau-Bouvery and Rodolakis, 2005; Glazunova et al., 2005; Svraka et al., 2006). A large number of MLVA data exists for European countries, even if the lack of consensus between scientists impedes comparison. Overall, a common pool of MLVA genotypes are present in Europe, together with novel genotypes sporadically found in specific countries (Astobiza et al., 2012; Santos et al., 2012; Ceglie et al., 2015; González-Barrio et al., 2016a; Joulié et al., 2017). Different MLVA genotypes could correspond to an identical MST type, indicating the MLVA method as more discriminatory than MST. MST genotyping reveals that the spacer types (ST)-8, ST-18, ST-20 are widespread over Europe, while other genotypes were found only in some countries (i.e. ST-32 in Greece and central Italy, ST-13 in Spain and Portugal, ST-4 in France and Portugal, ST-17 in French Guiana) (Glazunova et al., 2005; Chmielewski et al., 2009; Astobiza et al., 2012; Reichel et al., 2012; Santos et al., 2012; Di Domenico et al., 2014; Sulyok et al., 2014; Ceglie et al., 2015; Chochlakis et al., 2018).

Following the Dutch outbreak, a single nucleotide polymorphism (SNP) typing scheme was developed with the advantage of being a rapid method, providing however an intermediate discriminatory power. A panel of 10 SNPs was selected: 7 located in single copy genes and 3 in the multicopy gene *IS1111* (Huijsmans et al., 2011). Successively, other SNP schemes were developed based on the identification of polymorphisms present in MST loci or in intragenic conserved regions (Hornstra et al., 2011; Karlsson et al., 2014).

In Belgium, the prevalence of *C. burnetii* remained fairly unknown until 2009, when the Dutch outbreak pushed the Belgian authorities to initiate active surveillance of Q fever in small ruminants and cattle farms. From December 2009 to March 2013, goat farms located in 6 out of 10 Belgian provinces were positive for *C. burnetii* (Boarbi et al., 2014). MLVA and SNP genotypic types of strains in goat (Mori et al., 2013; Boarbi et al., 2014; Dal Pozzo et al., 2015) and SNP genotypes in cattle samples (Dal Pozzo et al., 2015) provided an initial fragmented viewpoint of Q fever in animals. However, there was a complete lack of genetic data in sheep and other animal species as well as an absolute absence of genotypic information from clinical human cases. To assess the likely animal sources important for human contamination in Belgium and to advance a One Health viewpoint of Q fever in the country, this work investigated genetic characteristics and spatial segregation of *C. burnetii* strains based on MLVA, SNP and whole genome sequence (WGS) data arising from strains cultured in axenic, cell culture and egg environments. Genotyping results and clinical presentation of human cases are presented and discussed, together with the large set of data obtained in different animal species. We showed that Q fever is widespread in Belgium and we identified *C. burnetii* as an abortive agent in alpaca, which has never been detected before.

## Materials and Methods

### Sample Collection

The prevalence analysis included samples from the abortion official program (total analyzed samples N=40,457 from cattle, N=349 from goats, N=1,310 from sheep) that is based on the mandatory/voluntary report of abortions that are successively analyzed for the presence of *C. burnetii.* Along this program, collected samples consists of placenta, cotyledon, vaginal swabs, fetal stomach content, fetal spleen, liver or any other relevant samples. The prevalence study incorporated also alpaca samples (N=33) retrieved from routine passive surveillance of farms in reference settings and tested for the presence of *C. burnetii*.

The genotyping study included Q fever positive samples from the abortion official program (N=103 from cattle, N=2 from goats, N=11 from sheep), bulk tank milks (BTM) from the BTM monitoring program (N=22 from goats, N=4 from sheep), alpaca sample from routine passive surveillance (N=1) and biological human samples (N=5 cardiac valves, aortic aneurism and blood) analyzed in the context of human National Reference Centre activities.

### Culturing of *C. burnetii*

*C. burnetii* strains were isolated from infected animal samples using two different strategies, including (1) *in vivo* isolation in OF1 mice (Charles Rivers, Wilmington, MA) and amplification in embryonated chicken eggs as previously described (Mori et al., 2017); (2) *in vitro* propagation in Vero cells followed by axenic cultivation in Acidified Citrate Cysteine Medium-2 (ACCM-2) as previously reported (Omsland et al., 2008; Omsland et al., 2009). Both procedures were adopted as strategy (1) was the only one available when strain isolation started. In addition, not all strains were able to grow in ACCM-2 (Kersh et al., 2016), therefore strategy (1) must be used for certain isolates.

### DNA Extraction and Diagnostic Real-Time PCR

DNA was extracted either directly from field samples for MLVA and SNP genotyping, or from *C. burnetii* culture (embryonated egg or axenic culture) for whole genome sequencing (WGS) studies. DNA was extracted from clinical human and veterinary samples with the MagMax™ Isolation Kit (Applied Biosystems; Thermo Fisher Scientific, Inc.) according to the manufacturer’s instructions. DNA was extracted directly from 200 µl of BTM, or homogenized abortion material or human samples (max. 1 gr in 1 ml MilliQ water). Next, 1/10^th^ of the eluted DNA was analyzed by real-time PCR targeting the *IS1111* repetitive element for the detection of *C. burnetii* as previously described (Klee et al., 2006; Mori et al., 2013). Results of the real-time PCR assays, performed with the 7500 Real-Time PCR System (Applied Biosystems; Thermo Fisher Scientific, Inc.) or Light cycler^®^ 480 Instrument II (Roche Molecular Systems, Inc., US), were expressed as cycle threshold (Ct) values. Samples displaying a Ct-value < 40 were considered to be positive.

DNA derived from embryonated eggs or axenic culture used for the WGS was obtained with the silica-based column method of the DNeasy Blood & Tissue Kit (Qiagen^®^, Hilden, Germany) from 200 µl sample following the manufacturer’s instructions. DNA was analyzed for the presence of *C. burnetii* by real-time PCR as described above. The total DNA quality and concentration was assessed by Nanodrop 1000 (Isogen Life Science, De Meern, Netherlands) measurements.

### MLVA and SNP Genotyping

DNA extracted directly from human and animal samples was analyzed for MLVA and SNP typing. We refer to these data as *in vitro* data. DNA positive samples having a Ct-value ≤ 30 were used for MLVA typing performed for 13 markers (MS3, MS12, MS21, MS22, MS30, MS36, MS23, MS24, MS27, MS28, MS31, MS33, MS34) (Arricau-Bouvery and Rodolakis, 2005; Svraka et al., 2006; Tilburg et al., 2012). Amplicons were analyzed by capillary electrophoresis on a CEQ 8000 Genetic Analysis System (Beckman Coulter, Indianapolis, IN, USA). The fragment sizes were defined by comparison to a 600 bp internal standard (Beckman Coulter, Indianapolis, IN, USA). Only MS12, whose unit size (126 bp) is not compatible with capillary electrophoresis, was run on 1% agarose gel electrophoresis and the PCR product size was determined according to a 100 bp DNA-ladder (Invitrogen; Thermo Fisher Scientific, Inc.). The number of repeats in each marker was established by extrapolating the sizes of the obtained amplicons relative to those obtained from the Nine Mile strain (RSA 493), used as a reference control strain. For MS33, readjustments of marker units were additionally operated following WGS analysis. The SNP typing was performed on *C. burnetii* positive DNA samples by real time PCR as previously reported (Huijsmans et al., 2011).

### Genotype Clustering and Geographical Distribution

MLVA and SNP data were analyzed using BioNumerics version 6.6 software (Applied Maths, Belgium). Minimum spanning trees were built on categorical data to assess the genetic relationship between *C. burnetii* profiles. MLVA analyses were performed on Belgian data (this study) and on public data, available from the MLVA online database^1^ and published in the literature. The geographical distribution of Belgian profiles was determined using the online LocalFocus Go tool^2^.

### WGS Sequencing and *In Silico* Genotyping

Only DNA extracted from embryonated eggs or axenic culture was used for WGS, since the DNA quantity deriving directly from field samples was insufficient for sequencing. WGS was performed on Illumina MiSeq platform using standard protocols (Garcia-Graells et al., 2020). For sequences obtained from embryonated eggs, read quality and filtering was calculated by ShortRead 1.16.331 package (Morgan et al., 2009) (Bioconductor^3^) and FastX 0.0.1333 (FastX-toolkit^4^), while adapters were trimmed with cutadapt 1.2 (Martin, 2011). PhiX and Gallus gallus 4.0 (galGal4) contaminants were removed using bowtie 2.0.0-beta5 (78.65% and 70.05% of reads were removed for the bovine CbBEB1 and the caprine CbBEC1 genomes, respectively). *De novo* assembly of processed pair reads was executed into CLC Genomics Workbench version 6.0.2. For sequences obtained from axenic cultures, read quality control and trimming were performed with FastQC (Galaxy Version 0.72) and Trimmomatic (Galaxy Version 0.38.0). *De novo* assembly was realized using SPAdes (Galaxy Version 3.12.0).

Contigs were subsequently analyzed for *in silico* genotyping. We refer to these data as *in silico* data. *In silico* MLVA typing was achieved for the 13 markers described above using Primersearch tool (Galaxy Version 5.0.0.1), which indicated the amplicon size corresponding to specific primer pairs of each marker. *In silico* SNP typing was performed *via* CLC Sequence Viewer version 8.0 using the probe/primer sequences described in Huijsmans et al. (2011) for the Dutch SNP panel, in Karlsson et al. (2014) for the canonical SNP (CanSNP) panel of intragenic region, in Hornstra et al. (2011) for the classification based on the CanSNP panel of MST loci. For the *in silico* MST, 10 primer pairs (Cox2, Cox5, Cox18, Cox20, Cox22, Cox 37, Cox51, Cox56, Cox57, Cox61) described in Glazunova et al. (2005) were used to identify the corresponding spacer sequences *via* CLC Sequence Viewer version 8.0. Spacer types were then assigned by blasting the obtained sequences against all *C. burnetii* spacers collected in the *C. burnetii* MST database^5^.

### Statistical Analyses

Prevalence data and 95% confidence interval (C.I.) were calculated using EPITOOLS^6^. The Hunter-Gaston discriminatory Index (HGDI) was calculated using Comparing Partitions tool^7^ to estimate the discriminatory power of individual VNTR loci (Hunter and Gaston, 1988). To determine differences between and within clusters, the analysis of similarity (ANOSIM) (Clarke, 1993) was carried out using Primer-e Software Version 7.

## Results

### Human Cases of Q Fever

In this study, we present five PCR-positive human cases associated with chronic (patient 1 to 4) and acute (patient 5) Q fever. Clinical presentations consisted of endocarditis, vascular infections and fever (the characteristics are summarized in **Table 1**). Three cases (patients 1 to 3) were submitted for diagnosis because of a blood culture negative endocarditis, while in one (patient 4) symptoms were related to a persistent abdominal aortic aneurysm. Endocarditis from patient 2 was discovered during a valve replacement following an aortic valve stenosis. In one case (patient 5), positive PCR result were detected in blood. The patient presented asthenia, irregular and nocturnal episodes of fever and pancytopenia. Positive serology was detected in four out of five patients (patient 1,2,4,5; data not shown). The most likely region of infection of all patients was determined based on medical request information and/or practitioner/patient interviews by local public health services. All cases where the likely contamination occurred in Belgium were associated with a SNP1 genotypic type, and MLVA clusters B5, B6 and B7. This genotype is closely related to the genotype of the strain responsible for the Dutch epidemic. The genotype of patient 4 belonged to SNP6/MLVA cluster C2 differing in repeat units size in MS22, MS36 and MS33 from similar strains found in Belgium.

**Table 1:** List of human Q fever cases.

ID	Month/Year of diagnosis	Most likely location of contamination	Risk factors	Clinical presentation	SNP genotype	MLVA cluster
**1 (2016/30)**	March 2016	Gent	Unknown	Endocarditis	SNP1	B5
**2 (2016/73)**	April 2016	Antwerp	Sheep farmer	Endocarditis	SNP1	B7
**3 (U1808763)**	September 2018	Namur	Unknown	Endocarditis	SNP1	B6
**4 (U1909659)**	October 2019	Unknown (imported)	Non-professional contact with animals, refugee	Abdominal aortic aneurysm	SNP6	C2
**5 (U2008815)**	September 2020	Liege	Unknown	Fever, asthenia, pancytopenia	SNP1	NA

NA, No available result.

### Animal Cases of Q Fever

Samples from the abortion official program provided data to estimate apparent prevalence of Q fever cases linked with abortion in cattle between 2011 and 2015 (after 2015, Q fever was no more systematically tested in all cattle abortive samples) and in sheep and goats between 2010 and 2019 (**Table 2**). The apparent prevalence of *C. burnetii* in bovine abortion samples was 1.93% (95% CI: 1.80%–2.07%), with an annual incidence ranging from 1.26% to 2.84% (N on average=8091). The highest incidence was observed in 2014. Beyond these years, *C. burnetii* positive rate in bovine could not be estimated due to the interruption of the program and a drastic decrease in sampling (<100 per year). *C. burnetii* apparent prevalence in goats was 9.19% (95% CI:6.59%–12.69%), with a range of annual incidence between 0%–17.65% (N on average=35) and a peak in the year 2019. Prevalence in sheep was 5.50% (95% CI: 4.39%–6.87%), reaching similar annual incidences as in goats 0-18.34% and a peak in 2017. Interestingly, among the passive surveillance samples, two cases of abortion positive to *C. burnetii* were detected in alpaca (*Vicugna pacos*). Differential diagnosis excluded *Toxoplasma gondii*, *Staphylococcus* sp., *Candida lambica*, *Brucella* sp., *Campylobacter* sp., *Chlamydia* sp., fungi which for all was negative. One of the two alpaca samples could be included in this genotyping study.

**Table 2:** Abortion cases recorded between 2010 and 2019 in cattle, goats, sheep, and alpacas.

Year/Species		2010	2011	2012	2013	2014	2015	2016	2017	2018	2019
Cattle	Total (N)	–	8,730	9,203	7,877	8,721	5,926	–	–	–	–
	Positive (N)	–	191	116	103	248	122	–	–	–	–
	Prevalence^a^ (%)	–	2.19	1.26	1.31	2.84	2.06	–	–	–	–
Goat	Total (N)	22	31	35	18	47	19	13	53	59	51
	Positive (N)	2	3	5	2	1	0	1	5	4	9
	Prevalence^a^ (%)	9.09	9.68	14.29	11.11	2.13	0.00	7.69	9.43	6.78	17.65
Sheep	Total (N)	60	112	377	61	85	116	49	169	158	123
	Positive (N)	2	1	1	2	4	0	2	31	19	10
	Prevalence^a^ (%)	3.33	0.89	0.27	3.28	4.71	0.00	4.08	18.34	12.03	8.13
Alpaca	Total (N)	1	–	–	–	3	8	1	7	5	8
	Positive (N)	0	–	–	–	0	0	0	1	0	1
	Prevalence^a^ (%)	NA	NA	NA	NA	NA	NA	NA	NA	NA	NA

^a^ Prevalence is given as apparent prevalence.

NA, Not applicable due to small sample size.

In addition, this study included goat and sheep BTM positive cases, gathered during the BTM surveillance program (2009-ongoing). In this case, *C. burnetii* positive rate has been described elsewhere (Boarbi et al., 2014).

### Molecular Phylogeny of Belgian *C. burnetii* Strains

**Figure 1:**
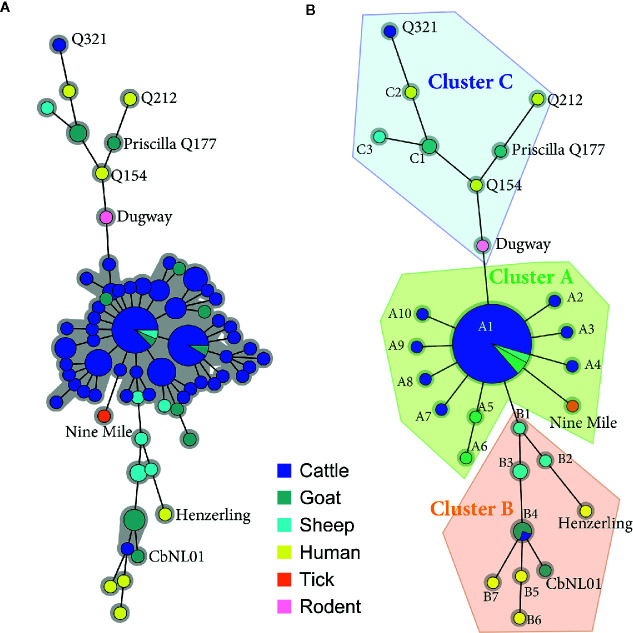
Minimum spanning trees showing the relationship between Multiple Locus Variable-number Tandem Repeat Analysis (MLVA) genotypes of Belgian isolates collected between 2010 and 2019. **(A)** Animal and human positive clinical samples with a complete 13-locus MLVA profile or missing max two out of 13 markers (n=114) were included in the clustering together with the reference strain profiles (n=8) obtained from published data (Roest et al., 2011; Joulié et al., 2017). Each circle corresponds to a single MLVA profile and the size of the circle is proportional to the number of samples sharing an identical genotype. The gray background connects samples differing of only one marker from each other. Homogeneous bovine and caprine genotypes are grouped in the core of the tree, while heterogeneous caprine and human profiles occupy the two lateral branches. **(B)** Minimum spanning tree resulting from the collapsing of **(A)** in which nodes differing of only one marker were merged together, as they may represent microvariants of the same genotype. This analysis separated 13-locus MLVA profiles in three main clusters (A, B, C) and different sub-clusters (A1-A12, B1-B8, C1-C3).

MLVA and SNP analysis were conducted to provide a comprehensive viewpoint of *C. burnetii* strains responsible for animal and human infections in Belgium. All human samples were characterized at the genetic level because they were extremely rare and difficult to obtain, by contrast only positive animal samples with a Ct value ≤ 30 were selected for the genetic characterization. Above this threshold, the genetic analysis could be unsuccessful due to a low bacterial DNA load. A total of 146 C*. burnetii* positive samples were included for MLVA typing with 13 markers (MLVA13) (**Table S1**). Only samples with a complete MLVA13 profile, or missing maximum two out of 13 markers, were used to build up the minimum spanning tree (n=114) and to evaluate genetic relationships between strains. The Unweighted Pair Group Method with Arithmatic Mean (UPGMA)-generated minimum spanning tree graph (**Figure 1A**), including references strains (n=8), depicted a central group including most of the cattle samples, some goat and sheep samples and giving origin to two smaller divisions represented by other goat, sheep samples and all human strains. Interestingly, within the central group no human samples were present. To identify specific phylogenic patterns, the minimum spanning tree built on the MLVA13 data was collapsed to include strains with similarity above 90% (only one marker difference). This resulted in a more compact tree divided in three clusters (A, B, C) and different sub-clusters (A1-A12, B1-B8, C1-C3) validated by the ANOSIM results (R = 0.851, p = 0.1%).

The subcluster A1 included most of the cluster A samples (91%), indicating the presence of relatively homogeneous profiles within cluster A. By contrast, the collapsing did not change sample distribution within clusters B and C, confirming their heterogeneity and division in separate subclusters.

**Figure 2:**
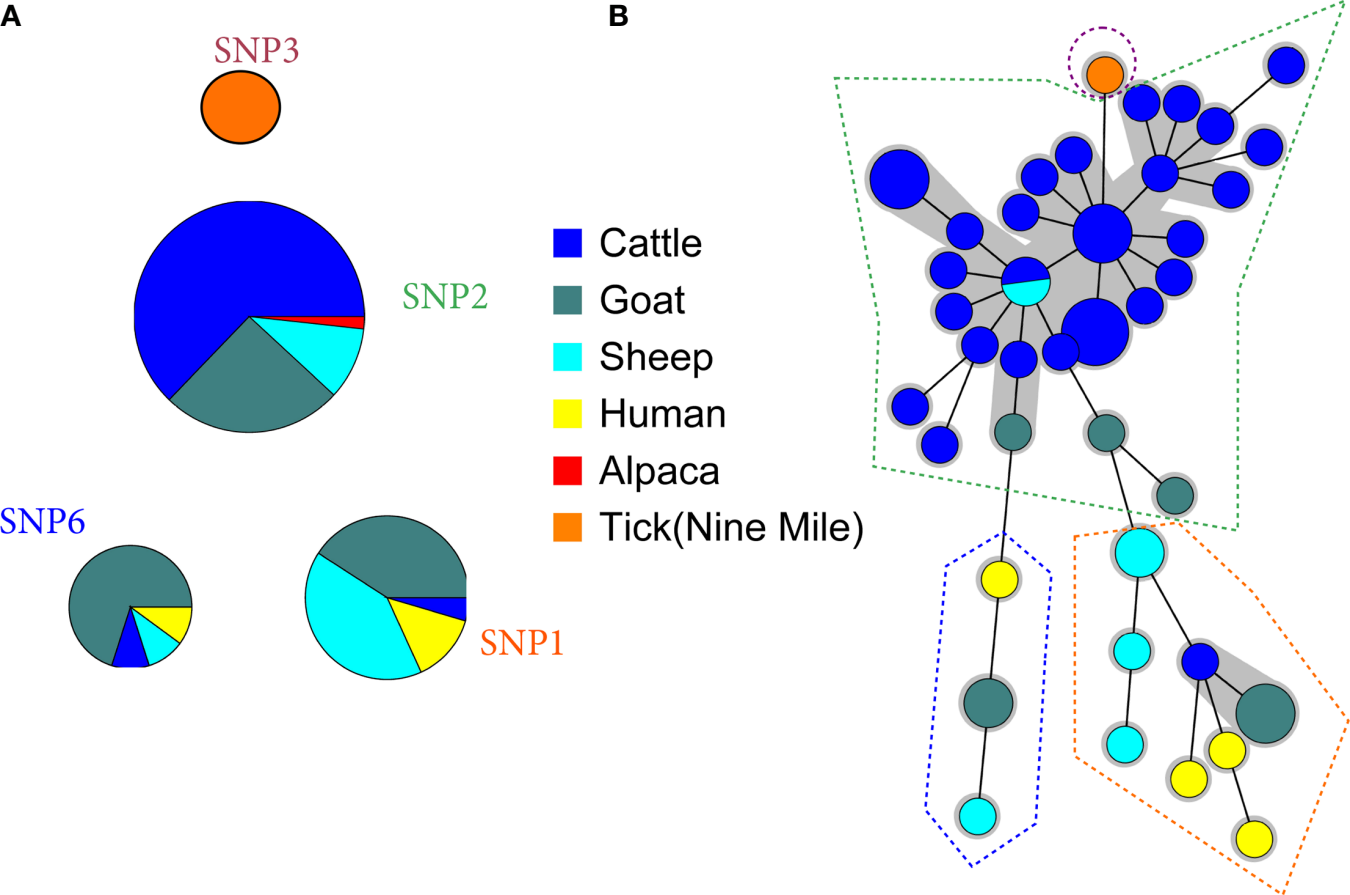
Comparison between **(A)** Single-Nucleotide Polymorphism (SNP) and **(B)** Multiple Locus Variable-number Tandem Repeat Analysis (MLVA) clustering. **(A)** Ninety-three Belgian clinical samples positive for *C. burnetii* were clustered basing on their SNP profile. **(B)** Fifty-two samples typed by SNP and MLVA were clustered by minimum spanning tree built on 13-locus MLVA profiles (complete or lacking max. two markers). MLVA profiles corresponding to a unique SNP genomic group are surrounded by a dotted line. Each circle corresponds to a single MLVA profile and the size of the circle is proportional to the number of samples sharing an identical genotype. The gray background connects samples differing of only one marker from each other.

In parallel, a total of 93 samples were characterized by SNP typing, revealing the presence of 3 major genotypes over the different years in Belgium: SNP1, SNP2, and SNP6 (**Figure 2A** and **Table S2**). The Nine Mile reference strain was confirmed as SNP3 type. The SNP2 profile prevailed upon Belgian small and big ruminant samples and defined the alpaca genotype. No human strain belonged to this genomic group, just as in the MLVA cluster A. By contrast, the SNP1 and SNP6 types were mainly detected in goat, sheep and human samples with a single case of abortion in cattle associated with an SNP1 profile, and one milk cattle sample presenting a SNP6 profile. To compare the MLVA and SNP typing, 52 samples characterized by both methods were used to construct two minimum spanning trees (**Figure 2**): one using SNP data (**Figure 2A**) and the other one using MLVA13 data (**Figure 2B**). **Figure 2B** showed that the SNP divisions were preserved among the MLVA classification, with however increased discriminant power conferred by the MLVA method. The HGDI calculated for individual VNTR loci, established that the discriminant power of MLVA panel II was significantly higher than MLVA panel I (**Table S3**). In addition, the HGDI (**Table S4**) highlighted that the SNP2 genotype matched to homogenous MLVA profiles (HDGI = 0.121), with only MS34 most subject to variations. By contrast, the SNP1 type was linked to heterogeneous MLVA profiles (HDGI = 0.399), with variation in the microsatellite panel. The SNP6 types mostly variated in three minisatellite markers (MS22, MS30, MS36) and one microsatellite marker (MS23). In addition, several MLVA profiles often missed the MS12, MS23, and MS33 markers (~18%, ~20% and ~30% of missing values, respectively) (**Table S1**).

### Geographical Distribution of Belgian *C. burnetii* Strains

**Figure 3:**
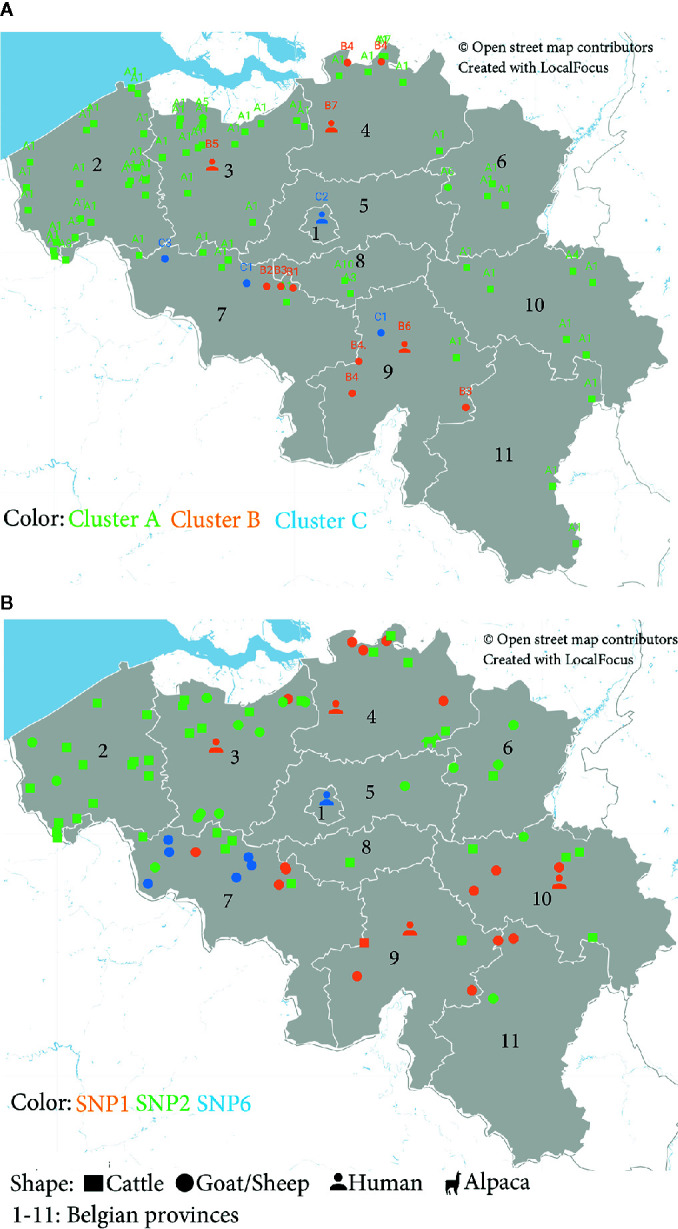
Geographical distribution of Belgian *C. burnetii* strains based on **(A)** Multiple Locus Variable-number Tandem Repeat Analysis (MLVA) and **(B)** Single-Nucleotide Polymorphism (SNP) genotypes detected between 2010 and 2019. **(A)** Localization of the MLVA profiles according to the clusters identified in **Figure 1B** and to the host species. **(B)** Distribution of *C. burnetii* positive samples according to the SNP genotypes (**Table S2**) and the host species.

The geographical distribution of *C. burnetii* strains circulating in Belgium was assessed by both MLVA and SNP data. Plotting MLVA clusters and subclusters on the Belgian map linked phylogeny with geographical distribution of each strain with a high degree of discriminatory power. The geographical localization of the MLVA profiles displayed that cluster A was dispersed over the territory, although it was particularly concentrated in the West Flanders (2) and East Flanders (3) provinces (56%) (**Figure 3A**). Cluster B was randomly present in all Belgian areas, while cluster C appeared more concentrated in the Western provinces (75%).

The geographical distribution of the Belgian *C. burnetii* strains based on the SNP classification confirms that the SNP6 (cluster C) genomic group is more localized in the Hainaut province (7) (89%), situated in the Western part of the country (**Figure 3B**).

### Phylogeny of European *C. burnetii* Strains

**Figure 4:**
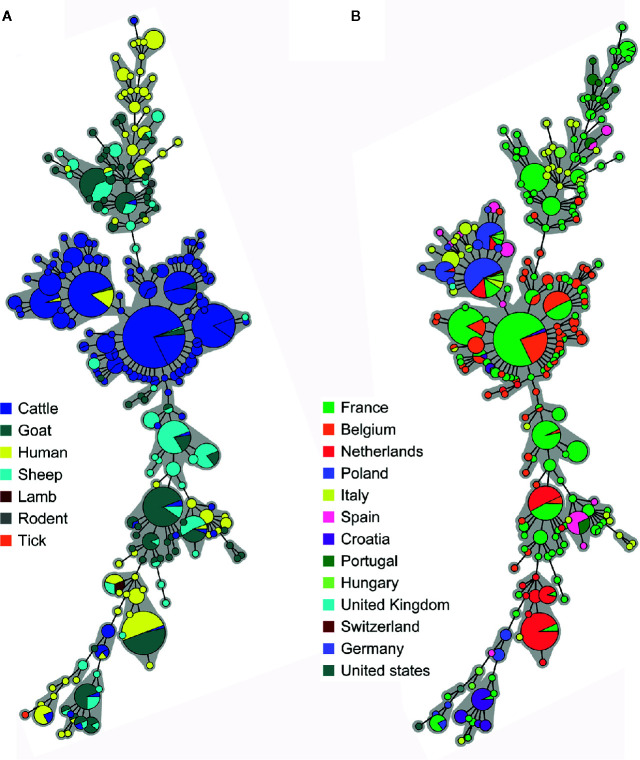
Minimum spanning trees showing the clustering of 855 Multiple Locus Variable-number Tandem Repeat Analysis (MLVA) profiles from European positive samples according to **(A)** the host species and **(B)** the country of origin. Data were collected from the present study, the MLVA online database and published studies. The microsatellite 7-locus MLVA panel was used for the clustering including only complete profiles, or missing max. two out of seven markers. Each circle corresponds to a single MLVA profile and the size of the circle is proportional to the number of samples sharing an identical genotype. The gray background connects samples differing of only one marker from each other.

Considering that live animals are traded every day in Europe, we examined the relationship between Belgian and other European (EU) *C. burnetii* strains. A database containing 955 MLVA profiles from 13 European countries was created assembling data from the present study, the MLVA online database and published articles (**Table S5**). Due to the lack of uniformity in the choice of markers, the comparison of all European profiles was challenging. For this reason, together with a large MLVA profiling (MLVA12, **Figure S1**), an MLVA7 analysis was performed to include more countries, and giving a broader view on the EU *C. burnetii* phylogeny (**Figure 4**). In the two analyses, complete profiles or those missing maximum 2 marker values, were used to build minimum spanning trees grouped by species (**Figure 4A** and **Figure S1A**) and by countries (**Figure 4B** and **Figure S1B**).

Most of the European cattle strains were grouped together in a central section, including Belgian caprine strains. Some French human samples from the 1990s were present as well. Strains from caprine, human and other animals (bull, rodent, lamb) were split into two lateral groups (**Figure 4A**). The division in three main clusters described for the Belgian strains could be extended to France, Italy, Spain, Portugal and Hungary as they presented samples in all three clusters. By contrast, Dutch, Polish and Croatian samples were limited to the central and one lateral group, indicating the possibility of two major genomic groups (**Figure 4B**). The CbNL01 strain, responsible for the Dutch outbreak, was present in Dutch, Belgian and French samples.

### WGS of Belgian *C. burnetii* Strains

In order to have a deeper and resolute view about *C. burnetii* genetic characterization, the genome of four Belgian isolates was sequenced (**Table 3**). Considering the complexity to sequence DNA directly from field samples, strains were first cultured, using the egg amplification strategy and/or the axenic culture isolation method, and successively sequenced. Isolates were selected with the aim of getting a representative bovine and/or caprine genome for each Belgian genetic group, although strain sequencing strongly depended on the success of culturing, which is extremely difficult for *C. burnetii* (Kersh et al., 2016). Two bovine (CbBEB1, CbBEB2) and two caprine (CbBEC1, CbBEC2) isolates were successfully cultured, sequenced and further analyzed for the *in silico* genotyping. All sequences presented an intact lipopolysaccharide (LPS) DNA sequence which characterizes virulent Phase I strains (data not shown), contrary to avirulent phase II bacteria which possess truncated LPS.

**Table 3:** Comparison between *in silico* and *in vitro* genotyping profiles of *C. burnetii* isolates sequenced in this study.

Typing Method	Isolate name	Host	Country	MLVA Markers - Number of repeats													SNP type
				MS03	MS12	MS21	MS22	MS30	MS36	MS27	MS28	MS31	MS23^*d*^	MS24	MS33	MS34	
*In silico-egg^c^*	CbBEC1	Goat	Belgium	7	8	6	6	11	13	3	3	3	NA	NA	8	7	1
*In vitro^b^*				7	8	6	6	11	13	3	3	3	NA	11	NA	7	1
*In silico-ACCM^a^*	CbBEB1	Cattle	Belgium	6	7	6	6	12	4	2	7	3	5	13	9	10	2
*In silico-egg^c^*				6	7	6	6	12	4	2	7	3	5	13	9	10	2
*In vitro^b^*				6	7	6	6	12	4	2	7	3	5	14	9	9	2
*In silico-ACCM^a^*	CbBEB2	Cattle	Belgium	6	7	6	6	12	4	2	7	3	5	13	9	9	2
*In vitro^b^*				6	7	6	6	12	4	2	7	3	5	13	9	9	2
*In silico-ACCM^a^*	CbBEC2	Goat	Belgium	6	7	6	6	12	4	2	7	3	5	13	9	10	2
*In vitro^b^*				6	7	6	6	12	4	2	7	3	NA	13	9	11	2

^a^ In silico genotyping of isolates sequenced after isolation by in vitro propagation in Vero cells followed by axenic cultivation in ACCM-2.

^b^ In vitro genotyping of isolates directly from positive animal samples.

^c^ In silico genotyping of isolates sequenced after in vivo isolation in OF1 mouse strain and amplification in embryonated chicken eggs.

^d^ Coding convention according to Arricau-Bouvery et al. (2006).

NA, Not available due to unsuccessful typing.

*In silico* SNP typing confirmed *in vitro* SNP results, with the CbBEB1, CbBEB2 and CbBEC2 strains belonging to the SNP2 type, while the CbBEC1 strain belonged to the SNP1 type. The *in silico* MLVA profiles of the sequenced strains were congruous with the *in vitro* results, except for three values (corresponding to MS24 marker for CbBEB1 and MS34 marker for CbBEB2 and CbBEC2), located in the microsatellite panel (**Table 3**).

The sequence of these isolates allowed *in silico* MST-typing and to establish for the first time the spacer types (ST) corresponding to the Belgian *C. burnetii* strains. Cattle and goat SNP2 isolates belonged to ST61, while the goat SNP1 strain was classified as ST33.

Sequences were also grouped using the CanSNP panel in MST loci which confirmed that the caprine SNP1 strain was close to the human and goat reference strains, while the SNP2 isolates were classified along with other ruminant reference strains (**Table 4**). Additionally, by comparison with published phylogeny (Hornstra et al., 2011; Hemsley et al., 2019), the SNP2 isolates were classified as genomic group III, while the SNP1 isolate was assigned to the genomic group II as described in Hendrix et al. (1991). The CanSNP panel in the intragenic region did not allow a separation between SNP2 and SNP1 isolates, therefore it did not allow to discriminate between genomic group III and II (**Table S6**).

**Table 4:** *In silico* analysis of sequenced isolates including Multispacer Sequence Typing (MST) typing and Canonical Single Nucleotide Polymorphism (CanSNP) in MST loci.

Isolate name	Host	Country	Year	CanSNP in MST loci														Branch*^c^*		MST type		Predicted Genomic group	
				Br.I.001 Cox18bp376	Br.I.003 Cox51bp563	Br.IV.001 Cox18bp34	Br.III.001 Cox5bp109	Br.IV.011 Cox22bp118	Br.II.007 Cox51bp492	Br.IV/VI Cox57bp327	Br.III.003 Cox56bp10	Br.II.004 Cox37bp215	Br.IV.015 Cox51bp67	Br.VI.001 Cox20bp155	Br.II.001 Cox18bp166	Br.V.001 Cox5bp81	Br.I/II/III Cox22bp91						
Nine Mile*^a^*	Tick	USA	1935	A	T	T	T	C	G	G	T	G	T	G	G	G	T	/		16		I	
Henzerling*^a^*	Human blood	Italy	1945	G	G	T	T	C	G	G	T	G	T	G	C	G	T	Br.II.001		18, 25		II	
CbBEC1	Goat milk	Belgium	2010	G	G	T	T	C	A	G	T	G	T	G	C/G	G	T	Br.II.007		33		II*^b^*	
CB2*^a^*	Human blood	France	/	G	G	T	T	C	A	G	T	G	T	G	C	G	T	Br.II.007		11–15, 24, 32-34		II	
CbBEB1	Cow abortion material	Belgium	2010	G	G	T	C	C	G	G	C	G	T	G	G	G	T	BrIII.001 & 003		61		III*^b^*	
CbBEB2	Cow stomach content	Belgium	2014	G	G	T	C	C	G	G	C	G	T	G	G	G	T	BrIII.001 & 003		61		III*^b^*	
CbBEC2	Goat milk	Belgium	2010	G	G	T	C	C	G	G	C	G	T	G	G	G	T	BrIII.001 & 003		61		III*^b^*	
CM-CA1*^a^*	Cow milk	California, USA	2007	G	G	T	C	C	G	G	C	G	T	G	G	G	T	BrIII.001 & 003		20		III	
CM-SC1*^a^*	Cow milk	South Carolina, USA	2007	G	G	T	C	C	G	G	C	G	T	G	G	G	T	BrIII.001 & 003		20		III	
ES-FL1*^a^*	Soil	Florida, USA	2010	G	G	T	C	C	G	G	C	G	T	G	G	G	T	BrIII.001 & 003		20		III	
Idaho goat*^a^*	Goat placenta	Idaho, USA	1981	G	G	T	C	C	G	G	C	G	T	G	G	G	T	BrIII.001 & 003		20		III	

^a^Genetic profile (CanSNP, MST type, predicted genomic group) from Hornstra et al. (2011) for comparison with isolates from this study.

^b^Predicted by comparison with phylogeny from Hornstra et al. (2011) based on Hendrix et al. (1991) genomic groups.

^c^It indicates the phylogenetic location(s) of each SNP on the phylogenetic tree from Hornstra et al. (2011).

## Discussion

### Q Fever in Human and Animal Cases

Q fever is a worldwide zoonosis caused by infection with *C. burnetii*. Domestic ruminants are the principal sources of human contamination (de Bruin et al., 2012), and there are suggestions that wild animal might occasionally play a role too (Jado et al., 2012; González-Barrio et al., 2016a). Consequently, it is important to understand the transmission dynamics within a specific epidemiological context and to tackle the likely routes from reservoir animals critical for the public health. In this study, Q fever was investigated and detected in all domestic ruminants and in humans.

In all, but one human case the disease was chronic, with the major clinical manifestation associated with endocarditis, which generally represents 60%–70% of all Q fever chronic cases (Brouqui et al., 1993; Maurin and Raoult, 1999). One case was due to vascular infection, that together with osteomyelitis, hepatitis, encephalitis and other rarer symptoms, represents the minor manifestations of the chronic form of the disease (Hatchette and Marrie, 2001; Manchal et al., 2020). The last case was a febrile patient with a positive PCR result in blood, indicating a probable recent involvement with the disease. The patient presented also pancytopenia, a rare manifestation of acute Q fever.

In animals, *C. burnetii* was detected in abortion of domestic ruminant species at different degrees of apparent prevalence. In bovine samples, *C. burnetii* annual incidence remained stable over the years (from 1.26% to 2.84%); by contrast, in goat and sheep abortion products, it was more susceptible to fluctuations (from 0% to ~18%). Of note, the sample size of the bovine samples was bigger than the caprine/ovine ones (thousand samples for bovine, hundred samples for caprine/ovine), highlighting the importance of large sample sizes for a more accurate estimation of *C. burnetii* prevalence. Differences in monitoring programs, sampling strategy and testing methods impedes a clear comparison of prevalence data among different countries and the estimation of Q fever true prevalence in Europe and worldwide (Guatteo et al., 2011; The European Union One Health 2018 Zoonoses Report, 2019).

In this study, we found *C. burnetii* in abortion material of alpaca. To the best of our knowledge, this is the first report of association of abortion with a positive PCR result on Q fever in alpaca. Infection of camelids by *C. burnetii* represents an important issue in several countries (i.e. Egypt, Algeria, Iran, Kenia, Saudi Arabia), where considerable levels of sero-prevalence (Benaissa et al., 2017; Browne et al., 2017; Khalafalla et al., 2017; Mobarez et al., 2017; Klemmer et al., 2018) and some abortion cases in camels were described (El-Deeb et al., 2019), but none has previously been observed in alpaca. Investigation on the role of *C. burnetii* in abortion in these animals and their potential role as reservoir for human contamination deserve certainly attention in the future.

### Genotyping and Geographical Distribution of Strains

*C. burnetii* molecular typing defined the genotypes circulating in Belgium and highlighted the relationship between animal and human strains, assessing the risk for human infection. MLVA and SNP typing methods were used to have a comprehensive viewpoint of Q fever in animals and humans through samples obtained from 2010 to 2019.

Overall, three MLVA clusters (A, B, C) subdivided in 23 subclusters (A1-A12, B1-B8, C1-C3) and three SNP types (SNP1, SNP2, SNP6) were identified. The high discriminative power conferred by the MLVA method was crucial for the identification of subclusters and accomplished an in-depth analysis. Although the SNP2 type/MLVA cluster A was confirmed to be the most abundant genotype in Belgium, it seemed not responsible for the human cases considered in this study, as it was only present in ruminant samples. However, interspecies transmission and maintenance of this genotype seemed to be high as it was found in samples from cattle, goat, sheep and alpaca. The SNP1/MLVA B and SNP6/MLVA C clusters were mostly found in small ruminant and human samples, with the (extremely rare) possibility in cattle. Similar incidental finding was observed also during the Q fever outbreak in the Netherlands (Huijsmans et al., 2011; Roest et al., 2013). The heterogeneous population structure in clusters B and C suggests that these strains could bear a higher capacity to evolve, possibly linked with the improved ability to infect more host species, including humans. Increased genome plasticity and mutations in membrane proteins and predicted virulence-associated genes were indeed identified in the CbNL01 strain, identical to our SNP1/MLVA cluster B strains (Kuley et al., 2017). Based on the overall results, we suggest a genomic group-host specificity, pointing at SNP1 and SNP6 genomic groups as responsible for human infections in Belgium. Both genotypes may cause not only chronic, as demonstrated here, but also acute Q fever (Huijsmans et al., 2011).

In Dal Pozzo et al. (2015) a novel genotype SNP5 was reported in Belgium, which was not confirmed in the present study. Noteworthy, Dal Pozzo et al. (2015) reported only a single sample with this genotype, which differs in a single marker from SNP1 type. Detection in a larger set of samples and/or isolation of the strain is warranted to confirm the circulation of this genotype in Belgium.

Regarding the MLVA data, as others, we observed a lack of data for MS23 and MS33 markers, especially in small ruminant samples by both *in vitro* and *in silico* MLVA typing. This suggests that also in Belgian strains an increased variability or mutations occurs in these regions, hampering their detection by the two methods (PCR and Illumina sequencing). The instability of these VNTR markers was previously proposed (Arricau-Bouvery et al., 2006; Svraka et al., 2006) and the unfeasibility to establish the number MS23 and/or MS33 repeats by *in vitro* or *in silico* MLVA was confirmed in other studies (Tilburg et al., 2012; Ceglie et al., 2015; Di Domenico et al., 2018). The presence of the recognition site for the insertion element *IS1111* in front of the repeat units of MS23 and MS33 makes these markers easy targets for *IS1111* insertion (Sidi-Boumedine et al., 2015).

To acquire a broader view on the MLVA types of *C. burnetii* strains present in Europe, we created adatabase containing 955 MLVA profiles from 13 European countries grouping published data or those present in available databases. The minimum spanning trees built on these MLVA profiles revealed the presence of 3 main groups: a central one including most of the cattle samples, and two lateral groups containing small ruminants and human samples, reflecting the distribution described for Belgium and in Joulié et al. (2017). The Nine Mile (tick) strain was located at the extremity of the trees, indicating an important phylogenetic distance with the European field strains. Considering that this strain is used for the production of the Coxevac^®^ vaccine employed for ruminant prevention, it raises the question of whether it is the best option to protect ruminant herds against Q fever.

In the European population structure, Belgian MLVA profiles are close to French and Dutch genotypes. Indeed, the SNP1, SNP2, and SNP6 types were also found in France and in the Netherlands (Huijsmans et al., 2011). In the Netherlands, the SNP1 and SNP6 groups were associated with human and goat samples (cattle spillover was present as well), while the SNP2 type only included animal strains (Huijsmans et al., 2011), supporting our hypothesis on the likely sources for the public health risk. As also indicated in Mori et al. (2013), the MLVA profile of SNP1 goat samples from 2010 were identical to the CbNL01 strain responsible for the Dutch outbreak. Three of these samples came from Antwerp, a region bordering the Netherlands, and one from the Namur province, situated at the Franco-Belgian border. The origin of the Namur sample is uncertain, although the owner could probably have exchanged animals with the bordering countries or within the country. Internal animal trade could be also responsible for strain transfer inside the Belgian territory. Of note, there has been an evolution of this genotype over time. In 2012, one sample from the Namur area presented an identical CbNL01 MLVA profile except for a single marker (MS36). Over the years, positive samples differed from the Dutch strain in more than one MLVA marker, keeping yet the SNP1 type features, and spreading all over the territory. It is possible that, as during the Dutch outbreak (Tilburg et al., 2012), the expansion of the *C. burnetii* SNP1 genotype in Belgium is occurring as well. The SNP2 group was also distributed throughout the country, even if Dal Pozzo et al. (2015) detected SNP2 bovine samples (2011–2014) mostly in the southern provinces of Belgium. This was probably due to the localization of the farms visited during that study. Instead, the SNP6 type was concentrated in the Hainaut and Namur provinces, two regions bordering France, confirming previous studies (Mori et al., 2013; Dal Pozzo et al., 2015).

### *In Silico* Genotyping

This study presented additionally WGS of four *C. burnetii* isolates originated from Belgium. Three genomes (the CbBEB1, CbBEB2, CbBEC2) were obtained from SNP2 animal samples (two cattle and one goat, respectively), while an additional one (the CbBEC1) corresponded to a goat SNP1 sample. The *in silico* genotyping of these isolates was used to complete our genomic study.

For the first time, the spacer types corresponding to *C. burnetii* Belgian strains were identified by *in silico* MST. Cattle and goat MLVA A/SNP2 isolates belonged to ST61. This is a new genotype only recently detected in Polish dairy cattle herds (Szymańska-Czerwińska et al., 2019). The goat MLVA B/SNP1 strain was ST33. This ST was also found in human and small ruminant samples (rare in cattle) from France, German and the Netherlands, and it was recently described in goat isolates from the UK (Chochlakis et al., 2018; Hemsley et al., 2019). Samples were additionally classified using two *in silico* CanSNP typing methods. The CanSNP in intragenic region was based on the identification of SNPs present in conserved region of the core genome (Karlsson et al., 2014). It was created as an alternative to the CanSNP in intergenic MST regions (Hornstra et al., 2011), considered as highly variable regions. In this study, the CanSNP in intragenic region method was unable to discriminate between SNP1 and SNP2 samples. By contrast, we could classify our samples using the CanSNP in MST regions. This classification confirmed that the caprine SNP1 strain was close to human and goat reference strains, while the SNP2 isolates were classified along with other ruminant reference samples. In addition, it was possible to predict the genomic groups (Hendrix et al., 1991) by comparison with the published phylogeny (Hornstra et al., 2011; Hemsley et al., 2019). The SNP1 strain (or ST33) belonged to the genomic group IIb, which included human, goat and sheep isolates (Hemsley et al., 2019). The SNP2 strain (or ST61) was part of the genomic group III, mainly composed by big ruminant samples (Hemsley et al., 2019). European isolates were predominant in the genomic group IIb and III (Hemsley et al., 2019). The genomic group III included also ST20, a genotype not found in Belgium, but present in several European countries (UK, France, Germany, Netherland, Italy, Spain, Hungary) (Glazunova et al., 2005; Astobiza et al., 2012; Reichel et al., 2012; Tilburg et al., 2012; Di Domenico et al., 2014; Sulyok et al., 2014). The ST20 was principally detected in cattle, rarely in small ruminants, and involved in farm animal outbreak (Reichel et al., 2012). During the Dutch outbreak, this strain type was only associated with animal samples and not with human infection (Tilburg et al., 2012; Roest et al., 2013). Nevertheless, a few ST20 human cases were detected in France in the 1990s (Glazunova et al., 2005). Interestingly, the minimum spanning tree that we created assembling MLVA European data, presented a central group including most of the cattle strains and a few French human samples from 1990s, suggesting that the association of humans with this genotype could be ascribed to a particular situation during that time frame.

The *in silico* genotyping analysis assessed the reliability of the *in vitro* MLVA and SNP results confirming that the SNP1 type is related to human and small ruminant infections, contrary to the SNP2 type that is mostly linked to animal infections.

In conclusion, the public health risk in Belgium appears to be linked to specific genomic groups and not to strains found predominantly in cattle. These conclusions are supported by studies in other European countries (Jado et al., 2012), although a limited number of human samples were applied here. The fact that human cases are more genetically linked to small ruminants strains could be attributed to the different manifestation of Q fever in the ruminant species (abortion storms-and therefore the release of enormous amount of infected material in the environment- in goats and sheep, sporadic cases in cattle) or to the intrinsic genetics of the strains. In either case, interspecies transmission of caprine/ovine genotypes can incidentally occur in cattle (i.e. SNP1 found in a rare case of bovine abortion). In these situations, exposure to cattle might be a risk for human health, therefore regular genotyping surveillance in the bovine sector should be promoted. These considerations could be extended to other European countries, especially those bordering Belgium, because of the concordance between Belgian and other European data as discussed above.

## Data Availability Statement

The datasets presented in this study can be found in online repositories. The names of the repository/repositories and accession number(s) can be found below: https://www.ncbi.nlm.nih.gov/, SAMN16591806, SAMN16591807, SAMN16591808, SAMN16591832, SAMN16591833.

## Ethics Statement

The approbation of the Bioethics Committee was not required in this study because data accessed here had not been collected for research purposes but as part of the routine data collection for epidemiological surveillance in accordance with article 18 of the law of 08/12/1992 of the Belgian Government regarding the protection of the privacy of the individual when dealing with personal data.

## Author Contributions

ST and MM defined conceptualization and methodology of the work. SB, FG, JC, DF, and MM organized sample collection. ST, SB, TF, PM, DD, and MM realized the tests. ST performed data curation and visualization. ST, SB, DF, EC, BD, and MM interpreted results. MM was responsible for funding acquisition and supervision. ST and MM wrote the manuscript. SB, DF, BD, and EC reviewed the manuscript. All authors contributed to the article and approved the submitted version.

## Funding

The NRC activity is supported by the Belgian Ministry of Social Affairs through a fund of the Health Insurance System. This work was partly supported by the project RF 10/6228 of the Federal Public Service of Health, Food Chain Safety and Environment. The Federal Agency for the Safety of the Food Chain (FASFC) funded the *C. burnetii* detection during the official surveillance programs in animals.

## Conflict of Interest

The authors declare that the research was conducted in the absence of any commercial or financial relationships that could be construed as a potential conflict of interest. 

## Footnotes

1
http://microbesgenotyping.i2bc.paris-saclay.fr/

2
https://go.localfocus.nl

3
http://www.bioconductor.org

4
http://hannonlab.cshl.edu/fastx_toolkit/index.html

5
https://ifr48.timone.univ-mrs.fr/mst/coxiella_burnetii/blast.html

6
https://epitools.ausvet.com.au/

7
http://www.comparingpartitions.info/

## References

[B1] Agerholm J. S. (2013). *Coxiella burnetii* associated reproductive disorders in domestic animals-a critical review. Acta Vet. Scand. 55, 13. 10.1186/1751-0147-55-13 23419216 PMC3577508

[B2] Arricau Bouvery N. Souriau A. Lechopier P. Rodolakis A. (2003). Experimental *Coxiella burnetii* infection in pregnant goats: excretion routes. Vet. Res. 34, 423–433. 10.1051/vetres:2003017 12911859

[B3] Arricau-Bouvery N. Rodolakis A. (2005). Is Q fever an emerging or re-emerging zoonosis? Vet. Res. 36, 327–349. 10.1051/vetres:2005010 15845229

[B4] Arricau-Bouvery N. Hauck Y. Bejaoui A. Frangoulidis D. Bodier C. C. Souriau A.. (2006). Molecular characterization of *Coxiella burnetii* isolates by infrequent restriction site-PCR and MLVA typing. BMC Microbiol. 6, 38. 10.1186/1471-2180-6-38 16640773 PMC1488860

[B5] Astobiza I. Tilburg J. J. H. C. Piñero A. Hurtado A. García-Pérez A. L. Nabuurs-Franssen M. H.. (2012). Genotyping of *Coxiella burnetii* from domestic ruminants in northern Spain. BMC Vet. Res. 8, 241. 10.1186/1746-6148-8-241 23227921 PMC3528428

[B6] Beare P. A. Samuel J. E. Howe D. Virtaneva K. Porcella S. F. Heinzen R. A. (2006). Genetic Diversity of the Q Fever Agent, Coxiella burnetii, Assessed by Microarray-Based Whole-Genome Comparisons. J. Bacteriol. 188, 2309–2324. 10.1128/JB.188.7.2309-2324.2006 16547017 PMC1428397

[B7] Benaissa M. H. Ansel S. Mohamed-Cherif A. Benfodil K. Khelef D. Youngs C. R.. (2017). Seroprevalence and risk factors for *Coxiella burnetii*, the causative agent of Q fever in the dromedary camel (*Camelus dromedarius*) population in Algeria. Onderstepoort J. Vet. Res. 84, e1–e7. 10.4102/ojvr.v84i1.1461 PMC6238797 28893076

[B8] Berri M. Souriau A. Crosby M. Crochet D. Lechopier P. Rodolakis A. (2001). Relationships between the shedding of *Coxiella burnetii*, clinical signs and serological responses of 34 sheep. Vet. Rec. 148, 502–505. 10.1136/vr.148.16.502 11345992

[B9] Boarbi S. Mori M. Rousset E. Sidi-Boumedine K. Van Esbroeck M. Fretin D. (2014). Prevalence and molecular typing of *Coxiella burnetii* in bulk tank milk in Belgian dairy goats 2009-2013. Vet. Microbiol. 170, 117–124. 10.1016/j.vetmic.2014.01.025 24598136

[B10] Brouqui P. Dupont H. T. Drancourt M. Berland Y. Etienne J. Leport C.. (1993). Chronic Q fever. Ninety-two cases from France, including 27 cases without endocarditis. Arch. Intern. Med. 153, 642–648. 10.1001/archinte.153.5.642 8439227

[B11] Browne A. S. Fèvre E. M. Kinnaird M. Muloi D. M. Wang C. A. Larsen P. S.. (2017). Serosurvey of *Coxiella burnetii* (Q fever) in Dromedary Camels (Camelus dromedarius) in Laikipia County, Kenya. Zoonoses Public Health 64, 543–549. 10.1111/zph.12337 28176495 PMC5655913

[B12] Ceglie L. Guerrini E. Rampazzo E. Barberio A. Tilburg J. J. H. C. Hagen F.. (2015). Molecular characterization by MLVA of *Coxiella burnetii* strains infecting dairy cows and goats of north-eastern Italy. Microbes Infect. 17, 776–781. 10.1016/j.micinf.2015.09.029 26526416

[B13] Chmielewski T. Sidi-Boumedine K. Duquesne V. Podsiadly E. Thiéry R. Tylewska-Wierzbanowska S. (2009). Molecular epidemiology of Q fever in Poland. Pol. J. Microbiol. 58, 9–13. 19469280

[B14] Chochlakis D. Santos A. S. Giadinis N. D. Papadopoulos D. Boubaris L. Kalaitzakis E.. (2018). Genotyping of *Coxiella burnetii* in sheep and goat abortion samples. BMC Microbiol. 18 (1), 204. 10.1186/s12866-018-1353-y 30514233 PMC6280429

[B15] Clarke K. R. (1993). Non-parametric multivariate analyses of changes in community structure. Aust. J. Ecol. 18, 117–143. 10.1111/j.1442-9993.1993.tb00438.x

[B16] Cutler S. J. Bouzid M. Cutler R. R. (2007). Q fever. J. Infect. 54, 313–318. 10.1016/j.jinf.2006.10.048 17147957

[B17] Dal Pozzo F. Renaville B. Martinelle L. Renaville R. Thys C. Smeets F.. (2015). Single Nucleotide Polymorphism Genotyping and Distribution of *Coxiella burnetii* Strains from Field Samples in Belgium. Appl. Environ. Microbiol. 82, 81–86. 10.1128/AEM.02799-15 26475104 PMC4702616

[B18] de Bruin A. van der Plaats R. Q. J. de Heer L. Paauwe R. Schimmer B. Vellema P.. (2012). Detection of *Coxiella burnetii* DNA on small-ruminant farms during a Q fever outbreak in the Netherlands. Appl. Environ. Microbiol. 78, 1652–1657. 10.1128/AEM.07323-11 22247143 PMC3298153

[B19] Di Domenico M. Curini V. De Massis F. Di Provvido A. Scacchia M. Cammà C. (2014). *Coxiella burnetii* in Central Italy: Novel Genotypes Are Circulating in Cattle and Goats. Vector Borne Zoonotic Dis. 14, 710–715. 10.1089/vbz.2014.1587 25325314 PMC4208599

[B20] Di Domenico M. Curini V. Di Lollo V. Massimini M. Di Gialleonardo L. Franco A.. (2018). Genetic diversity of *Coxiella burnetii* in domestic ruminants in central Italy. BMC Vet. Res. 14, 171. 10.1186/s12917-018-1499-8 29843709 PMC5975477

[B21] El-Deeb W. Ghoneim I. Fayez M. Elsohaby I. Alhaider A. ElGioushy M. (2019). Acute phase proteins, proinflammatory cytokines and oxidative stress biomarkers in sheep, goats and she-camels with *Coxiella burnetii* infection-induced abortion. Comp. Immunol. Microbiol. Infect. Dis. 67, 101352. 10.1016/j.cimid.2019.101352 31568899

[B22] Garcia-Graells C. Berbers B. Verhaegen B. Vanneste K. Marchal K. Roosens N. H. C.. (2020). First detection of a plasmid located carbapenem resistant blaVIM-1 gene in E. coli isolated from meat products at retail in Belgium in 2015. Int. J. Food Microbiol. 324, 108624. 10.1016/j.ijfoodmicro.2020.108624 32302878

[B23] Georgiev M. Afonso A. Neubauer H. Needham H. Thiery R. Rodolakis A.. (2013). Q fever in humans and farm animals in four European countrie to 2010. Euro. Surveill. 18 (8), 20407. 23449232

[B24] Glazunova O. Roux V. Freylikman O. Sekeyova Z. Fournous G. Tyczka J.. (2005). *Coxiella burnetii* genotyping. Emerg. Infect. Dis. 11, 1211–1217. 10.3201/eid1108.041354 16102309 PMC3320512

[B25] González-Barrio D. Hagen F. Tilburg J. J. H. C. Ruiz-Fons F. (2016a). *Coxiella burnetii* Genotypes in Iberian Wildlife. Microb. Ecol. 72, 890–897. 10.1007/s00248-016-0786-9 27216529

[B26] González-Barrio D. Jado I. Fernández-de-Mera I. G. Fernández-Santos M. del R. Rodríguez-Vargas M.. (2016b). Genotypes of *Coxiella burnetii* in wildlife: disentangling the molecular epidemiology of a multi-host pathogen. Environ. Microbiol. Rep. 8, 708–714. 10.1111/1758-2229.12431 27336914

[B27] Guatteo R. Beaudeau F. Berri M. Rodolakis A. Joly A. Seegers H. (2006). Shedding routes of *Coxiella burnetii in* dairy cows: implications for detection and control. Vet. Res. 37, 827–833. 10.1051/vetres:2006038 16973121

[B28] Guatteo R. Seegers H. Taurel A.-F. Joly A. Beaudeau F. (2011). Prevalence of *Coxiella burnetii* infection in domestic ruminants: a critical review. Vet. Microbiol. 149, 1–16. 10.1016/j.vetmic.2010.10.007 21115308

[B29] Hatchette T. F. Marrie T. J. (2001). Atypical Manifestations of Chronic Q Fever. Clin. Infect. Dis. 33, 1347–1351. 10.1086/323031 11565075

[B30] Hemsley C. M. O’Neill P. A. Essex-Lopresti A. Norville I. H. Atkins T. P. Titball R. W. (2019). Extensive genome analysis of *Coxiella burnetii* reveals limited evolution within genomic groups. BMC Genomics 20, 441. 10.1186/s12864-019-5833-8 31164106 PMC6549354

[B31] Hendrix L. R. Samuel J. E. Mallavia L. P. (1991). Differentiation of *Coxiella burnetii* isolates by analysis of restriction-endonuclease-digested DNA separated by SDS-PAGE. J. Gen. Microbiol. 137, 269–276. 10.1099/00221287-137-2-269 1673152

[B32] Hornstra H. M. Priestley R. A. Georgia S. M. Kachur S. Birdsell D. N. Hilsabeck R.. (2011). Rapid typing of *Coxiella burnetii* . PloS One 6, e26201. 10.1371/journal.pone.0026201 22073151 PMC3206805

[B33] Huijsmans C. J. J. Schellekens J. J. A. Wever P. C. Toman R. Savelkoul P. H. M. Janse I.. (2011). Single-nucleotide-polymorphism genotyping of *Coxiella burnetii* during a Q fever outbreak in The Netherlands. Appl. Environ. Microbiol. 77, 2051–2057. 10.1128/AEM.02293-10 21257816 PMC3067327

[B34] Hunter P. R. Gaston M. A. (1988). Numerical index of the discriminatory ability of typing systems: an application of Simpson’s index of diversity. J. Clin. Microbiol. 26, 2465–2466. 3069867 10.1128/jcm.26.11.2465-2466.1988 PMC266921

[B35] Jado I. Carranza-Rodríguez C. Barandika J. F. Toledo Á. García-Amil C. Serrano B.. (2012). Molecular method for the characterization of *Coxiella burnetii* from clinical and environmental samples: variability of genotypes in Spain. BMC Microbiol. 12, 91. 10.1186/1471-2180-12-91 22656068 PMC3413600

[B36] Joulié A. Sidi-Boumedine K. Bailly X. Gasqui P. Barry S. Jaffrelo L.. (2017). Molecular epidemiology of *Coxiella burnetii* in French livestock reveals the existence of three main genotype clusters and suggests species-specific associations as well as regional stability. Infect. Genet. Evol. 48, 142–149. 10.1016/j.meegid.2016.12.015 28007602

[B37] Karlsson E. Macellaro A. Byström M. Forsman M. Frangoulidis D. Janse I.. (2014). Eight New Genomes and Synthetic Controls Increase the Accessibility of Rapid Melt-MAMA SNP Typing of *Coxiella burnetii* . PloS One 9, e85417. 10.1371/journal.pone.0085417 24465554 PMC3897454

[B38] Kersh G. J. Priestley R. A. Hornstra H. M. Self J. S. Fitzpatrick K. A. Biggerstaff B. J.. (2016). Genotyping and Axenic Growth of *Coxiella burnetii* Isolates Found in the United States Environment. Vector Borne Zoonotic Dis. 16, 588–594. 10.1089/vbz.2016.1972 27304166 PMC5011622

[B39] Khalafalla A. I. Al Eknah M. M. Abdelaziz M. Ghoneim I. M. (2017). A study on some reproductive disorders in dromedary camel herds in Saudi Arabia with special references to uterine infections and abortion. Trop. Anim. Health Prod. 49, 967–974. 10.1007/s11250-017-1284-x 28364266

[B40] Klee S. R. Tyczka J. Ellerbrok H. Franz T. Linke S. Baljer G.. (2006). Highly sensitive real-time PCR for specific detection and quantification of. Coxiella Burnetii BMC Microbiol. 6, 2. 10.1186/1471-2180-6-2 16423303 PMC1360083

[B41] Klemmer J. Njeru J. Emam A. El-Sayed A. Moawad A. A. Henning K.. (2018). Q fever in Egypt: Epidemiological survey of *Coxiella burnetii* specific antibodies in cattle, buffaloes, sheep, goats and camels. PloS One 13, e0192188. 10.1371/journal.pone.0192188 29466380 PMC5821454

[B42] Kuley R. Kuijt E. Smits M. A. Roest H. I. J. Smith H. E. Bossers A. (2017). Genome Plasticity and Polymorphisms in Critical Genes Correlate with Increased Virulence of Dutch Outbreak-Related *Coxiella burnetii* Strains. Front. Microbiol. 8, 1526. 10.3389/fmicb.2017.01526 28848533 PMC5554327

[B43] Manchal N. Adegboye O. A. Eisen D. P. (2020). A systematic review on the health outcomes associated with non-endocarditis manifestations of chronic Q fever. Eur. J. Clin. Microbiol. Infect. Dis. 39 (12), 2225–2233. 10.1007/s10096-020-03931-7 32661808

[B44] Martin M. (2011). Cutadapt removes adapter sequences from high-throughput sequencing reads. EMBnet J. 17, 10–12. 10.14806/ej.17.1.200

[B45] Maurin M. Raoult D. (1999). Q fever. Clin. Microbiol. Rev. 12, 518–553. 10515901 10.1128/cmr.12.4.518 PMC88923

[B46] Mobarez A. M. Amiri F. B. Esmaeili S. (2017). Seroprevalence of Q fever among human and animal in Iran; A systematic review and meta-analysis. PloS Negl. Trop. Dis. 11, e0005521. 10.1371/journal.pntd.0005521 28394889 PMC5398711

[B47] Morgan M. Anders S. Lawrence M. Aboyoun P. Pagès H. Gentleman R. (2009). ShortRead: a bioconductor package for input, quality assessment and exploration of high-throughput sequence data. Bioinformatics 25, 2607–2608. 10.1093/bioinformatics/btp450 19654119 PMC2752612

[B48] Mori M. Boarbi S. Michel P. Bakinahe R. Rits K. Wattiau P.. (2013). In vitro and in vivo infectious potential of *coxiella burnetii*: a study on Belgian livestock isolates. PloS One 8, e67622. 10.1371/journal.pone.0067622 23840751 PMC3695903

[B49] Mori M. Mertens K. Cutler S. J. Santos A. S. (2017). Critical Aspects for Detection of *Coxiella burnetii* . Vector Borne Zoonotic Dis. 17, 33–41. 10.1089/vbz.2016.1958 28055578

[B50] Omsland A. Cockrell D. C. Fischer E. R. Heinzen R. A. (2008). Sustained axenic metabolic activity by the obligate intracellular bacterium *Coxiella burnetii* . J. Bacteriol. 190, 3203–3212. 10.1128/JB.01911-07 18310349 PMC2347409

[B51] Omsland A. Cockrell D. C. Howe D. Fischer E. R. Virtaneva K. Sturdevant D. E.. (2009). Host cell-free growth of the Q fever bacterium *Coxiella burnetii* . PNAS 106, 4430–4434. 10.1073/pnas.0812074106 19246385 PMC2657411

[B52] Piñero A. Barandika J. F. García-Pérez A. L. Hurtado A. (2015). Genetic diversity and variation over time of *Coxiella burnetii* genotypes in dairy cattle and the farm environment. Infect. Genet. Evol. 31, 231–235. 10.1016/j.meegid.2015.02.006 25684412

[B53] Reichel R. Mearns R. Brunton L. Jones R. Horigan M. Vipond R.. (2012). Description of a *Coxiella burnetii* abortion outbreak in a dairy goat herd, and associated serology, PCR and genotyping results. Res. Vet. Sci. 93, 1217–1224. 10.1016/j.rvsc.2012.04.007 22578355

[B54] Riley L. W. Blanton R. E. (2018). Advances in Molecular Epidemiology of Infectious Diseases: Definitions, Approaches, and Scope of the Field. Microbiol. Spectr. 6 (6). 10.1128/microbiolspec.AME-0001-2018 PMC6343655 30387413

[B55] Riley L. W. (2019). Differentiating Epidemic from Endemic or Sporadic Infectious Disease Occurrence *. Microbiol. Spectr. 7 (4). 10.1128/microbiolspec.AME-0007-2019 PMC10957193 31325286

[B56] Rodolakis A. Berri M. Héchard C. Caudron C. Souriau A. Bodier C. C.. (2007). Comparison of *Coxiella burnetii* shedding in milk of dairy bovine, caprine, and ovine herds. J. Dairy Sci. 90, 5352–5360. 10.3168/jds.2006-815 18024725

[B57] Roest H. I. J. Ruuls R. C. Tilburg J. J. H. C. Nabuurs-Franssen M. H. Klaassen C. H. W. Vellema P.. (2011). Molecular epidemiology of *Coxiella burnetii* from ruminants in Q fever outbreak, the Netherlands. Emerg. Infect. Dis. 17, 668–675. 10.3201/eid1704.101562 21470457 PMC3377418

[B58] Roest H. I. J. van Solt C. B. Tilburg J. J. H. C. Klaassen C. H. W. Hovius E. K. Roest F. T. F.. (2013). Search for possible additional reservoirs for human Q fever, The Netherlands. Emerg. Infect. Dis. 19, 834–835. 10.3201/eid1905.121489 23697680 PMC3647510

[B59] Rousset E. Berri M. Durand B. Dufour P. Prigent M. Delcroix T.. (2009). *Coxiella burnetii* shedding routes and antibody response after outbreaks of Q fever-induced abortion in dairy goat herds. Appl. Environ. Microbiol. 75, 428–433. 10.1128/AEM.00690-08 19011054 PMC2620711

[B60] Santos A. S. Tilburg J. J. H. C. Botelho A. Barahona M. J. Núncio M. S. Nabuurs-Franssen M. H.. (2012). Genotypic diversity of clinical *Coxiella burnetii* isolates from Portugal based on MST and MLVA typing. Int. J. Med. Microbiol. 302, 253–256. 10.1016/j.ijmm.2012.08.003 23040417

[B61] Sidi-Boumedine K. Duquesne V. Prigent M. Yang E. Joulié A. Thiéry R.. (2015). Impact of IS1111 insertion on the MLVA genotyping of *Coxiella burnetii* . Microbes Infect. 17, 789–794. 10.1016/j.micinf.2015.08.009 26342253

[B62] Stein A. Saunders N. A. Taylor A. G. Raoult D. (1993). Phylogenic homogeneity of *Coxiella burnetii* strains as determinated by 16S ribosomal RNA sequencing. FEMS Microbiol. Lett. 113, 339–344. 10.1111/j.1574-6968.1993.tb06537.x 7505761

[B63] Sulyok K. M. Kreizinger Z. Hornstra H. M. Pearson T. Szigeti A. Dán Á.. (2014). Genotyping of *Coxiella burnetii* from domestic ruminants and human in Hungary: indication of various genotypes. BMC Vet. Res. 10, 107. 10.1186/1746-6148-10-107 24885415 PMC4016735

[B64] Svraka S. Toman R. Skultety L. Slaba K. Homan W. L. (2006). Establishment of a genotyping scheme for *Coxiella burnetii* . FEMS Microbiol. Lett. 254, 268–274. 10.1111/j.1574-6968.2005.00036.x 16445755

[B65] Szymańska-Czerwińska M. Jodełko A. Zaręba-Marchewka K. Niemczuk K. (2019). Shedding and genetic diversity of *Coxiella burnetii* in Polish dairy cattle. PloS One 14, e0210244. 10.1371/journal.pone.0210244 30629637 PMC6328121

[B66] The European Union One Health 2018 Zoonoses Report. (2019). EFSA J. 17, e05926. 10.2903/j.efsa.2019.5926 32626211 PMC7055727

[B67] Tilburg J. J. H. C. Roest H. J. I. J. Nabuurs-Franssen M. H. Horrevorts A. M. Klaassen C. H. W. (2012). Genotyping reveals the presence of a predominant genotype of *Coxiella burnetii in* consumer milk products. J. Clin. Microbiol. 50, 2156–2158. 10.1128/JCM.06831-11 22495560 PMC3372143

[B68] To H. Htwe K. K. Kako N. Kim H. J. Yamaguchi T. Fukushi H.. (1998). Prevalence of *Coxiella burnetii* infection in dairy cattle with reproductive disorders. J. Vet. Med. Sci. 60, 859–861. 10.1292/jvms.60.859 9713817

[B69] Woldehiwet Z. (2004). Q fever (coxiellosis): epidemiology and pathogenesis. Res. Vet. Sci. 77, 93–100. 10.1016/j.rvsc.2003.09.001 15196898

