# Supplementary material for: Phylogeography of Human and Animal Coxiella burnetii Strains: Genetic Fingerprinting of Q Fever in Belgium
Source: Front Cell Infect Microbiol. 2021 Feb 26;10:625576. doi: 10.3389/fcimb.2020.625576 (PMC7952626; doi:10.3389/fcimb.2020.625576)
Supplement: Supplementary file 1 [file Image_1.pdf]

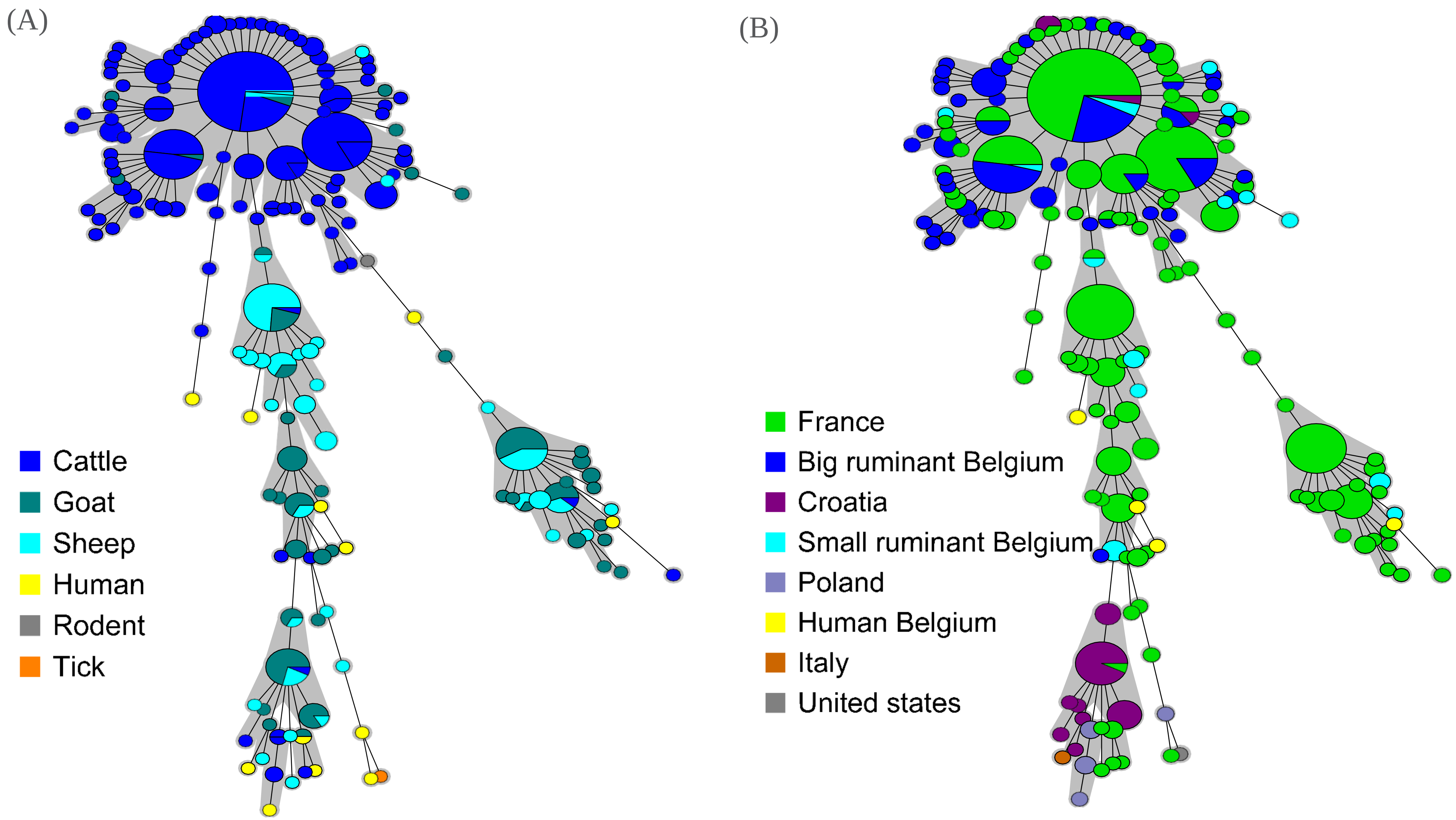

Supplementary Figure 1: Minimum spanning trees representing 12-locus MLVA profiles (N=449) from European positive samples clustered on (A) the host species and (B) the country of origin. Data came from Table S2 and includes complete profiles or missing max. two out of 12 markers (MS3, MS12, MS21, MS22, MS36, MS23, MS24, MS27, MS28, MS31, MS33, MS34). Each circle corresponds to single MLVA profile and the size of the circle is proportional to the number of samples sharing an identical genotype. The gray background connects samples differing for only one marker from each other.
