# Supplementary material for: Phylogeography of Human and Animal Coxiella burnetii Strains: Genetic Fingerprinting of Q Fever in Belgium
Source: Front Cell Infect Microbiol. 2021 Feb 26;10:625576. doi: 10.3389/fcimb.2020.625576 (PMC7952626; doi:10.3389/fcimb.2020.625576)
Supplement: Supplementary file 3 [file Table_2.pdf]

Supplementary Table 2: SNP genotypes of Belgian *C. burnetii* isolates detected in animal and human samples tested positive by qPCR between 2010 and 2020.

**Year**	**Host**	**Source**	**Motif**	**Nb. of samples**	**SNP type**
	Tick	Reference strain-Nine Mile		1	**3**
2010	Cattle	Abortion material, stomach content	Abortion	18	**2**
	Goat	Bulk tank milk	Screening	5	**1**
		Bulk tank milk	Screening	2	**2**
		Bulk tank milk	Screening	3	**6**
2011	Cattle	Abortion material, fetus	Abortion	2	**2**
2012	Cattle	Stomach content	Abortion	1	**2**
		Fetus	Abortion	1	**1**
	Goat	Stomach content	Abortion	1	**2**
2013	Cattle	Fetus	Abortion	1	**2**
2014	Cattle	Stomach content, fetus	Abortion	6	**2**
	Goat	Bulk tank milk	Screening	1	**1**
		Bulk tank milk	Screening	1	**6**
	Sheep	Bulk tank milk	Screening	1	**1**
2015	Cattle	Abortion material,	Abortion	7	**2**
		stomach content, fetus		1	**6**
		Individual milk	Screening		
	Goat	Bulk tank milk	Screening	3	**1**
		Bulk tank milk	Screening	1	**2**
2016	Cattle	Abortion material, Bulk tank milk	Screening	2	**2**
	Goat	Bulk tank milk	Screening	1	**1**
		Bulk tank milk	Screening	7	**2**
		Bulk tank milk	Screening	3	**6**
	Sheep	Individual milk	Screening	1	**1**
	Human	Biological material	Diagnosis	2	**1**
2017	Goat	Bulk tank milk	Screening	1	**1**
		Stomach content	Abortion	2	**2**
	Sheep	Abortion material, stomach content, spleen	Abortion	6	**1**
		Abortion, material stomach content, spleen	Abortion	6	**2**
	Alpaca	Stomach content	Abortion	1	**2**
2018	Sheep	Bulk tank milk	Screening	1	**1**
		Bulk tank milk	Screening	1	**6**
	Human	Biological material	Diagnosis	1	**1**
2019	Human	Biological material	Diagnosis	1	**6**
2020	Human	Biological material	Diagnosis	1	**1**
**Total**				**93**	
