# Supplementary material for: Phylogeography of Human and Animal Coxiella burnetii Strains: Genetic Fingerprinting of Q Fever in Belgium
Source: Front Cell Infect Microbiol. 2021 Feb 26;10:625576. doi: 10.3389/fcimb.2020.625576 (PMC7952626; doi:10.3389/fcimb.2020.625576)
Supplement: Supplementary file 4 [file Table_3.pdf]

Supplementary Table 3: Hunter Gaston diversity index (HGDI) for individual loci calculated from Belgian MLVA profiles.

	Marker type	No. of alleles	HGDI	CI (1) (95%)	Panel mean
Panel I	MS03	3	0.231	(0.147-0.315)	0.174*
	MS12	3	0.107	(0.033-0.180)	
	MS21	2	0.104	(0.037-0.170)	
	MS22	2	0.082	(0.020-0.144)	
	MS30	7	0.33	(0.235-0.424)	
	MS36	4	0.192	(0.099-0.285)	
Panel II	MS23	11	0.255	(0.151-0.360)	0.370*
	MS24	10	0.509	(0.416-0.602)	
	MS27	3	0.312	(0.219-0.405)	
	MS28	7	0.42	(0.323-0.516)	
	MS31	3	0.043	(1.000-0.090)	
	MS33	6	0.208	(0.105-0.312)	
	MS34	14	0.845	(0.806-0.883)	

(1) Confidence interval at 95%.

* T-Test between Panel I and Panel II ($p<0.05$).
