# Supplementary material for: Phylogeography of Human and Animal Coxiella burnetii Strains: Genetic Fingerprinting of Q Fever in Belgium
Source: Front Cell Infect Microbiol. 2021 Feb 26;10:625576. doi: 10.3389/fcimb.2020.625576 (PMC7952626; doi:10.3389/fcimb.2020.625576)
Supplement: Supplementary file 5 [file Table_4.pdf]

Supplementary Table 4: Hunter Gaston diversity index (HGDI) for individual loci calculated from Belgian MLVA profiles divided in SNP1, SNP2 and SNP6 types.

SNP type	Marker type	No. of alleles	HGDI	CI (1) (95%)	Mean
SNP1	MS03	2	0.366	(0.148-0.584)	0.399
	MS12	1	0	(0.000-0.000)	
	MS21	2	0.118	(1.000-0.319)	
	MS22	1	0	(0.000-0.000)	
	MS30	2	0.125	(1.000-0.337)	
	MS36	3	0.703	(0.628-0.779)	
	MS23	6	0.952	(0.855-1.000)	
	MS24	2	0.513	(0.388-0.637)	
	MS27	2	0.515	(0.430-0.599)	
	MS28	2	0.133	(1.000-0.357)	
	MS31	1	0	(0.000-0.000)	
	MS33	3	1	(0.456-1.000)	
	MS34	4	0.773	(0.645-0.900)	
SNP2	MS03	2	0.050	(1.000-0.144)	0.121*
	MS12	1	0	(0.000-0.000)	
	MS21	1	0	(0.000-0.000)	
	MS22	1	0	(0.000-0.000)	
	MS30	1	0	(0.000-0.000)	
	MS36	1	0	(0.000-0.000)	
	MS23	1	0	(0.000-0.000)	
	MS24	3	0.583	(0.517-0.649)	
	MS27	2	0.049	(1.000-0.140)	
	MS28	1	0	(0.000-0.000)	
	MS31	1	0	(0.000-0.000)	
	MS33	1	0	(0.000-0.000)	
	MS34	7	0.83	(0.781-0.878)	
SNP6	MS03	1	0	(0.000-0.000)	0.169*
	MS12	1	0	(0.000-0.000)	
	MS21	1	0	(0.000-0.000)	
	MS22	2	0.4	(1.000-0.829)	
	MS30	2	0.4	(1.000-0.829)	
	MS36	2	0.4	(1.000-0.829)	
	MS23	2	1	(1.000-1.000)	
	MS24	1	0	(0.000-0.000)	
	MS27	1	0	(0.000-0.000)	
	MS28	1	0	(0.000-0.000)	
	MS31	1	0	(0.000-0.000)	
	MS33	1	0	(0.000-0.000)	
	MS34	1	0	(0.000-0.000)	

(1) Confidence interval at 95%.

* T-Test against SNP1 group ($p < 0.05$).
