# Supplementary material for: Phylogeography of Human and Animal Coxiella burnetii Strains: Genetic Fingerprinting of Q Fever in Belgium
Source: Front Cell Infect Microbiol. 2021 Feb 26;10:625576. doi: 10.3389/fcimb.2020.625576 (PMC7952626; doi:10.3389/fcimb.2020.625576)
Supplement: Supplementary file 7 [file Table_6.pdf]

**Supplementary Table 6**: *In silico* canSNP typing in intragenic region of *Coxiella burnetii* isolates sequenced in this study.

| Isolate name | Host | Country | CanSNP in intragenic conserved region | | | | | | | | | | Derived for CanSNP[b] | SNP type |
|---|---|---|---|---|---|---|---|---|---|---|---|---|---|---|
| | | | C.1 | C.3 | C.4 | C.5 | C.6 | C.7 | C.8 | C.9 | C.10 | C.11 | | |
| Nine Mile | Tick | US | C | T | G | T | G | A | C | A | T | G | C10 | 3 |
| CbBEC1 | Cattle | Belgium | C | NA[c] | G | T | G | A | C | A | NA[c] | A | C11 | 1 |
| CbBEB1 | Cattle | Belgium | C | T | G | T | G | A | C | A | C | A | C11 | 2 |
| CbBEB2 | Goat | Belgium | C | T | G | T | G | A | C | A | C | NA[c] | C11 | 2 |
| CbBEC2 | Goat | Belgium | C | T | G | T | G | A | C | A | C | A | C11 | 2 |
| Henzerling[a] | Human | Italy | C | T | G | T | G | A | C | A | C | A | C11 | 8 |

a Genetic profile from *Karlsson et al.* (2014) for comparison with isolates from this study.
b Based on phylogeny from *Karlsson et al.* (2014).
c NA: No available result from MLVA typing.
